# 影像组学在肺癌精准诊疗中的研究进展

**DOI:** 10.3779/j.issn.1009-3419.2019.06.09

**Published:** 2019-06-20

**Authors:** 张 史

**Affiliations:** 影像组学在肺癌精准诊疗中的研究进展 Department of Radiology, Changhai Hospital, Sencond Military Mecdical University, Shanghai 200433, China

**Keywords:** 肺肿瘤, 影像组学, 精准医疗, Lung neoplasms, Radiomics, Precision medicine

## Abstract

精准医疗，影像先行。影像组学作为人工智能中机器学习的一种方法，通过肿瘤感兴趣区进行定量分析，提供有价值的诊断、预后或预测信息，以支持个性化的临床决策和改善个体化的治疗选择，为实现精准医疗的目标打下了坚实基础。本文将综述影像组学在肺癌精准诊断和治疗的最新研究进展。

## Abstract (en)

Precision medicine, imaging first. Radiomics, a method of machine learning in artificial intelligence, provides valuable diagnostic, prognostic or predictive information through quantitative analysis on the tumor's region of interest to support personalized clinical decisions and improve individualized treatment, which could lay a solid foundation for achieving the precision medicine. This review provides a latest advance of the radiomic application of the precision medicine for lung cancer.

肺癌是世界上最具侵袭性的癌症之一，其5年总生存率为10%-15%，在过去三十年中没有显著改善^[1]^。因此，精准诊治肺癌是临床医生一直追求的目标。精准诊治就是以精确诊断为前提、精确治疗为目的的精准诊疗体系，针对个体疾病进行分子水平分析和靶向技术治疗。目前，影像学方法是肺癌诊断及治疗的重要手段，并且能够预测治疗结果及评估治疗效果，而要实现精准诊治就必须突破基于形态学及半定量分析的传统影像医学模式^[2]^。影像组学（radiomics）作为人工智能（artificial intelligence, AI）中机器学习的一种方法为实现这一目标打下了坚实的基础，它融合了数字影像信息、统计学、机器学习等方法，采用高通量特征提取算法，对影像图像进行定量分析，最高效地利用影像学检查结果^[2]^。近几年来，肺癌一直处于影像组学研究的最前沿，包括基于计算机断层扫描（computed tomography, CT）、磁共振成像（magnetic resonance imaging, MRI）及正电子发射计算机断层显像（positron emission tomography/CT, PET/CT）等各种影像检查的影像组学特征，为肺癌的精准诊治开辟了新的道路。本文将综述影像组学在肺癌精准诊断和治疗的研究进展。

## 1 影像组学的内涵

影像组学是将影像定量分析与机器学习方法结合起来，它被认为是AI的一种形式。影像组学特征是定量图像特征，可以提供肿瘤的强度、形状、大小、体积以及纹理特征等。不同成像模式（如MRI、CT、PET、超声等）均可作为影像组学特征提取的基础^[3]^。从影像图像中提取的全部特征就是“影像组学”，而通过特征选择后所挑选出的那些具有预测价值的特征集合通常被称为“影像组学标签（radiomic signature）”^[2]^。目前，影像组学的基本作用是通过大量的影像组学特征对肿瘤感兴趣区进行定量分析，从而可以提供有价值的诊断、预后或预测信息。其目的是探索和利用这些信息资源来开发诊断、预测或预后的影像组学模型，以支持个性化的临床决策和改善个体化的治疗选择^[4]^。

## 2 影像组学的方法

影像组学的研究方法分为五个步骤^[5]^：①图像采集：主要通过CT、MRI和PET/CT等影像扫描方式来进行图像的采集。CT是影像组学研究中使用最广泛的成像模式^[4, 5]^；②图像分割：是指对感兴趣区部位的分割，也就是在影像图像上勾画出感兴趣区域，从而针对这一特定区域计算出影像组学特征。目前，图像分割有三种方法，即人工分割法、半自动分割法及自动分割法；③图像特征提取和量化：图像分割得到ROI后，就可以进行定量影像组学特征的提取和分析。影像组学特征可以分为形状特征、一阶直方图特征、二阶直方图或纹理特征。还有一些获取于特定图像的影像组学特征（如PET中的SUV度量）以及仅适用于多模式数据集的分形和融合特征；④特征选择：特征选择就是用一种算法来选择给定任务的“有效”特征，最简单的特征选择方法是根据变量的稳定程度或相关性制定一个评分标准，以此标准对变量进行筛选；⑤建立模型：影像组学的目标是根据预测结果和影像组学特征来开发一种新的功能或建立一个新的数学模型，以此对患者进行分类。

## 3 影像组学在肺癌精确诊疗中的应用

近十多年来，影像组学用于肿瘤的预测及分类的模型研究取得了长足进步，可用于肿瘤分期、病理分型、肺癌诊断、鉴别诊断、治疗方案选择、疗效监测及预后评估等多个方面。目前，影像组学在肺癌中的研究是开始最早、进行最广泛，且成果较多的研究应用，其中非小细胞肺癌（non-small cell lung cancer, NSCLC）的影像组学研究最为深入。

### 3.1 肿瘤分期

肿瘤分期对后续临床治疗方案选择有重要的作用。在一项基于PET/CT图像的122例NSCLC患者影像组学研究中，Flechsig等^[6]^通过直方图分析法，发现恶性淋巴结中位CT密度值比良性淋巴结明显增高，且两者有统计学差异（*P* < 0.05），该研究表明，对于FDG摄取不明显的肺癌患者，密度值测量可作为一个替代参数进行良恶性判别，并且可以无创地评估肺癌淋巴结分期。而2018年的一项研究^[7]^表明，影像组学的方法既可以用来进行肿瘤的分期，也可以准确地预测肿瘤分型，为后续临床精准诊疗提供帮助，该研究基于肺癌CT图像的影像组学特征，通过特征提取和建模，发现该方法不仅有助于区分腺癌、鳞癌和大细胞癌，而且还能预测淋巴结转移和远处转移情况。

### 3.2 病理分型

根据2015版世界卫生组织（World Health Organization, WHO）肺癌分类方法，上皮来源的肺癌病理分型可分为腺癌、鳞癌、神经内分泌肿瘤、大细胞癌、腺鳞癌等10种类型，其中常见分型主要为腺癌、鳞癌和大细胞癌。Ferreira-Junior等^[7]^基于肺癌CT图像的影像组学特征，发现该方法在区分腺癌、鳞癌和大细胞癌时训练组和验证组的AUC分别为0.71和0.81，表明影像组学方法在肺癌组织病理学亚型诊断中具有很大的潜力。在NSCLC的病理分型上，一项2018年关于NSCLC影像组学的研究^[8]^表明，通过LASSO *Logistic*回归模型建立的影像组学标签能够很好地区分腺癌和鳞癌，训练集和验证集的AUC分别为0.905和0.893。此外，Yuan等^[9]^分析了431例肺腺癌患者的CT图像，通过影像组学和体积分析法在区分肺腺癌不同病理表型中的比较，发现影像组学能够用于区分原位腺癌、微浸润腺癌和浸润性腺癌，并且预测准确率达到80.5%，明显高于常规的体积分析法。

### 3.3 肺癌诊断

在肺癌诊断方面，有研究^[11]^通过影像组学预测模型的构建，对良、恶性肺结节进行评估，且取得了较佳的效果。目前，影像组学用于预测肺结节良恶性的准确率可达76.10%^[11]^；其中，一项从593例肺癌CT图像中提取150个影像组学特征进行分析，发现该组学定量特征的训练集对肺癌诊断准确率可达86.0%，而验证集准确率为76.1%^[11]^。2017年的一项中国研究将肺结节恶性程度分为五级，基于影像组学特征及随机森林建模，发现单个肺结节的5类恶性程度平均预测准确率为77.85%，而对于每例患者肺结节恶性程度的预测准确率为75.16%^[12]^。另外，一项关于采用低剂量CT扫描方法诊断肺癌的研究表明影像组学也可用于肺癌的早期检测，对预测恶性结节有较大的价值，并且可以进一步评估肺癌发展的风险^[13]^。

### 3.4 鉴别诊断

一直以来，病理活检是诊断NSCLC的金标准，而影像组学通过无创、经济、便捷的方法，能够揭示肉眼不可见的特征，从而丰富并补充了肺部肿瘤鉴别诊断的方法。其中，Aerts等^[13]^发现基于CT扫描的影像组学特Laws-Energy与表皮生长因子受体（epidermal growth factor receptor, *EGFR*）突变型有高相关性，可以作为病理分型的重要预测因素。而另一项与EGFR相关的肺癌研究通过分析352例患者*EGFR*突变检测并结合CT图像资料，发现影像组学特征能有效区分EGFR野生型和*EGFR*突变型，该模型的AUC=0.69^[10]^。另外，准确区分肿瘤基因型也对临床精准治疗提供重要的帮助。2017年，一项通过人工智能深度学习方法的研究，采用自动量化的影像组学特征建立能区分肺癌基因型的影像组学标签，其结果表明利用影像组学建立的影像学标记显著优于常规影像学指标（肿瘤的体积、最大直径），能够较准确地判断*EGFR*的突变状态，因此，该研究认为影像组学可以预测不同基因的突变状态，且具有无创性、可重复性、价廉的优点^[15]^。

### 3.5 治疗方案选择

在治疗方案的选择上，Ohri等^[16]^从201名患者的多中心数据中发表了一个使用LASSO程序的影像组学模型，从GLCM计算出的一个纹理特征（Sum_Mean_）作为总体生存率的独立预测因子，结果表明，代谢肿瘤体积（metabolic tumor volume, MTV）的理想值为93.3 cm^3^，而相对应的Sum_Mean_值为0.018，当肿瘤为高MTV和低Sum_Mean_时，患者的总体生存率最低，进行化疗的效果差。近来，中国学者的一项基于治疗前MR-DWI图像对肺癌化疗治疗效果预测的影像组学分析结果表明影像组学模型对鉴别治疗有效组和无效组敏感度和特异性均较高，并且基于*b*=600 s/mm^2^、800 s/mm^2^和1, 000 s/mm^2^的AUC差异无统计学意义，该研究表明影像组学可以在治疗前预测肺癌化疗的疗效，为治疗方案选择提供新的角度^[17]^。

### 3.6 疗效监测

在肺癌的疗效监测上，根据治疗前后三个疗程之间的CT影像组学特征变化，影像组学识别集可以评估经吉非替尼（一种EGFR酪氨酸激酶抑制剂）治疗后患者的疗效情况，且两次扫描之间影像组学特征变化具有显著差异（特征的AUC为0.74-0.91）^[9]^。此外，Fave等^[18]^通过对治疗期间NSCLC的影像组学特征进行分析，发现定量影像组学特征可以预测总体生存、远处转移等，并且影像组学特征是肿瘤治疗反应的关键指标。

### 3.7 预后评估

在预后评估方面，一项关于通过增强和平扫CT来预测无病生存期的影像组学研究表明，基于增强CT的峰度、均质性和均一性以及平扫CT的均一性这4个组学特征所形成的影像组学识别集对患者无病生存期的预测效果优于用传统的肿瘤分期预测效果^[19]^。而另一项基于静态自由呼吸（free breathing, FB）和平均强度投影（average intensity projection, AIP）CT扫描图像的影像组学研究发现，AIP多变量影像组学模型在预测远处转移上优于其他所有模型，这可能与AIP图像所包含的信息与立体定向体部放疗（stereotactic body radiation therapy, SBRT）治疗早期NSCLC患者疾病复发相关^[20]^。此外，Lynch等^[21]^采用人工智能的大量监督学习技术，通过对肿瘤分级、大小、阶段和数量以及患者性别、年龄的初选，对肺癌患者的生存进行分类，最终结果显示，预测性能最好的技术是自定义集成，最精确的模型为梯度增强机（gradient boosting machine, GBM），而支持向量机（SVM）是唯一生成独特输出的模型，这一结果表明这些监督学习技术可以用来评估肺癌患者的生存时间。

## 4 影像组学的挑战

近年来影像组学的研究逐年增多，因其提取特征及建模的方法和形式具有多样性，而有研究表明目前影像组学所建立的预测模型质量不佳。有学者提出了影像组学质量评分（radiomics quality score, RQS）及其标准^[22]^，帮助我们科学评估之前做过的以及未来将要进行的影像组学研究。RQS标准对预测模型的所有方面都需要进行全面和清晰的评估和打分，以尽量减少偏差，从而提高预测模型的实用性。

影像组学是大数据时代的产物，充足的特征数据及构建数据库是影像组学研究前提。目前，许多影像组学研究大多是小样本量的研究^[23]^，而样本量不足所产生的小数据集会降低模型预测准确率并增加过度拟合的风险，因此影像组学依旧存在很多问题和挑战。

首先，数据的可重复性较低。可重复性是在相同或几乎相同的条件和采集参数下的精度测量，并通过“测试-重新测试”分析进行评估，比较对同一患者采集图像的结果。有研究^[24]^表明，在相同成像参数设置和半自动分割下获得的影像组学特征可重复性较高（一致性指数 > 0.9）。

其次，在特征提取和量化及建模方面，每项研究有不同的选择和不同的思路，大多数研究都是通过多种尝试及测量后找到最合适的一种特征提取和建模方法，但这种方式的人为选择性和随机性较大，而且在统计与数据库方面，大多数影像组学研究并没有在独立队列研究中得到充分验证，从而患者人群的普适性不高。

最后，标准化程度不高。大多数影像组学研究使用的图像是从多个研究机构的各种扫描方案或来自不同供应商的扫描仪。笔者认为需要通过扫描方案及重建算法的标准化来降低输入数据的变异度，尤其是多中心的研究更应如此。建议从影像组学研究开始就设定好一个方案，并按照RQS标准严格把控，从而提高研究质量。

## 5 影像组学的展望

影像组学尚处于起步阶段，虽然在临床疾病的诊断、疗效监测及预后评估中都取得了一些成果，但是影像组学依然有一些局限和不足，工作流程中也有可能改进的地方，并且在许多情况下仅仅是统计学上的差异，而不能给出明确的是与非。随着肺癌精准诊疗的大力倡导，影像组学以其低成本、无创、避免不必要的治疗和毒性风险等优点为个性化医疗提供了一种新的方法。未来，规范而标准的成像图像所提取的影像组学特征建立的数据库，将为我们提供一个精准诊疗的优质化医疗帮助，从而为实现精准医疗迈出坚实的一步。

## References

[1] Constanzo J, Wei L, Tseng HH (2017). Radiomics in precision medicine for lung cancer. Transl Lung Cancer Res.

[2] Shi Z, Li J, Bian Y (2018). The study progress of the radiomics in the clinical precision medicine. Zhonghua Fang She Xue Za Zhi.

[3] Parekh V, Jacobs MA (2016). Radiomics: a new application from established techniques. Expert Rev Precis Med Drug Dev.

[4] Gillies RJ, Kinahan PE, Hricak H (2016). Radiomics: Images are more than pictures, they are data. Radiology.

[5] Shi Z, Liu Q (2018). The study and challenge of radiomic techonlogy and method. Fang She Xue Shi Jian.

[6] Flechsig P, Frank P, Kratochwil C (2017). Radiomic analysis using density threshold for FDG-PET/CT-based N-staging in lung cancer patients. Mol Imaging Biol.

[7] Ferreira Junior JR, Koenigkam-Santos M, Cipriano FEG (2018). Radiomics-based features for pattern recognition of lung cancer histopathology and metastases. Comput Methods Programs Biomed.

[8] Zhu X, Dong D, Chen Z (2018). Radiomic signature as a diagnostic factor for histologic subtype classification of non-small cell lung cancer. Eur Radiol.

[9] Yuan M, Zhang YD, Pu XH (2017). Comparison of a radiomic biomarker with volumetric analysis for decoding tumour phenotypes of lung adenocarcinoma with different disease-specific survival. Eur Radiol.

[10] Jun W, Xia L, Di D (2016). Prediction of malignant and benign of lung tumor using a quantitative radiomic method. Conf Proc IEEE Eng Med Biol Soc.

[11] Yang CR, Guo Y, Wang YY (2017). Prediction of pulmonary nodule malignancy based on radiomics. Zhong Liu Ying Xiang Xue.

[12] Hawkins S, Wang H, Liu Y (2016). Predicting malignant nodules from screening CT scans. J Thorac Oncol.

[13] Aerts HJ, Grossmann P, Tan Y (2016). Defining a radiomic response phenotype: a pilot study using targeted therapy in NSCLC. Sci Rep.

[14] Hochhegger B, Zanon M, Altmayer S (2018). Advances in imaging and automated quantification of malignant pulmonary diseases: a state-of-the-art review. Lung.

[15] Rios Velazquez E, Parmar C, Liu Y (2017). Somatic mutations drive distinct imaging phenotypes in lung cancer. Cancer Res.

[16] Ohri N, Duan F, Snyder BS (2016). Pretreatment 18F-FDG PET textural features in locally advanced non-small cell lung cancer: secondary analysis of ACRIN 6668/RTOG 0235. J Nucl Med.

[17] Jiang JZ, Ding YY, LI ZH (2017). DWl-based radiomics for predicting response of lung cancer to chemotherapy: a pilot study. Fang She Xue Shi Jian.

[18] Fave X, Zhang L, Yang J (2017). Delta-radiomics features for the prediction of patient outcomes in non-small cell lung cancer. Sci Rep.

[19] Huang YQ, Liang CH, He L (2016). Development and validation of a radiomics nomogram for preoperative prediction of lymph node metastasis in colorectal cancer. J Clin Oncol.

[20] Huynh E, Coroller TP, Narayan V (2017). Associations of radiomic data extracted from static and respiratory-gated CT scans with disease recurrence in lung cancer patients treated with SBRT. PLoS One.

[21] Lynch CM, Abdollahi B, Fuqua JD (2017). Prediction of lung cancer patient survival via supervised machine learning classification techniques. Int J Med Inform.

[22] Lambin P, Leijenaar RTH, Deist TM (2017). Radiomics: the bridge between medical imaging and personalized medicine. Nat Rev Clin Oncol.

[23] Napel S, Giger M (2015). Special section guest editorial: radiomics and imaging genomics: quantitative imaging for precision medicine. J Med Imaging (Bellingham).

[24] Hatt M, Tixier F, Pierce L (2017). Characterization of PET/CT images using texture analysis: the past, the present, any future?. Eur J Nucl Med Mol Imaging.

